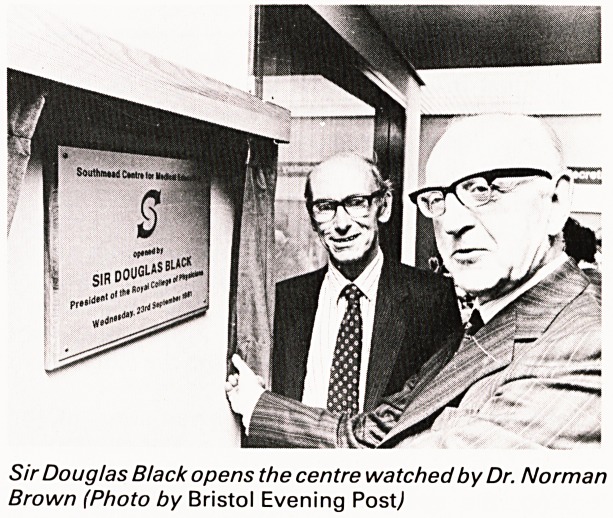# Southmead Centre for Medical Education

**Published:** 1982

**Authors:** 


### Southmead Centre for Medical Education

The newly-built postgraduate medical education centre at Southmead Hospital was officially opened on 23rd September 1981 by Sir Douglas Black, President of the Royal College of Physicians.

As an extension to the existing University medical school unit opened in 1972 the new building not only adds further lecture and tutorial rooms but also provides social, dining and office amenities to complete a functionally integrated teaching complex to be known as the Southmead Centre for Medical Education. It is believed that this is possibly the first time that such a unit, combining undergraduate and postgraduate teaching under one roof, has been created in a District Hospital in this country.

The new building has a total area of 471 sq. metres. The main lecture room seats 80 and is provided with the usual audio-visual facilities, including X-ray viewing boxes, blackout, dimming lights and video equipment. Having a flat floor and moveable seating it is highly adaptable and can be used, for example, for exhibitions. It will prove a useful adjunct to the Atholl Riddell lecture theatre in the older building. Adjacent to the lecture room a comfortable waiting room has been provided for use by patients and their attendants when clinical demonstrations are being held. The small seminar room seats 12 and again has suitable teaching aids. There is a large store for teaching equipment, such as display boards, and for valuable equipment when not actually being used. The dining room, which seats 60, is designed for self-service via the adjacent servery and kitchen. The dining room leads from the foyer area by glazed folding doors which can be thrown back for larger functions. In the foyer there is a bar, and large French windows lead from the foyer to an outside paved area. Offices for the secretary/receptionist, who will oversee the Centre, and the Clinical Tutors lead off the link corridor to the older teaching facilities which retain the medical library, tutorial and common rooms, University offices and laboratories and the medical illustration department.

The opening ceremony, attended by a large gathering of present and former hospital staff together with representatives of the University and other educational organisations and preceded by lunch was presided over by Dr. N. J. Brown, chairman of the District Medical Education Committee, who briefly recounted the development of medical education at Southmead commending the enthusiasm and dedication of the many people who despite grossly inadequate facilities had contributed so much time and effort over many years to postgraduate teaching. The postgraduate centre had been designed in almost exactly its present form in the 1950's when it was hoped it would be the first in the South Western Region. For a variety of reasons it was now in fact the last to be provided in the Region but for many at Southmead including himself, its opening was the fulfilment of a dream that had lasted for a quarter of a century.

*Sir Douglas Black opens the centre watched by Dr. Norman Brown (Photo by* Bristol Evening Post*)*

Dr. Brown then introduced Sir Douglas Black who in a most entertaining speech declared his affection for Bristol where, to mention only one connection, his daughter had been a medical student. He wished the Centre every success and unveiled a plaque commemorating the occasion.

A vote of thanks to Sir Douglas and Lady Black was proposed by Dr. T. L. Chambers, one of the Postgraduate Clinical Tutors, after which the guests were able to tour the new centre and view displays of work exhibited by the various clinical departments.

## Figures and Tables

**Figure f1:**